# An Expanding Large Acrochordon on the Labia Majora Requiring Surgical Intervention

**DOI:** 10.7759/cureus.79017

**Published:** 2025-02-14

**Authors:** Shahrokh Ahkami, Daniel Hahn, Hannah Fowler-Kim, Ron Jako Domingo

**Affiliations:** 1 Obstetrics and Gynecology, St. Mary's General Hospital, Passaic, USA; 2 Obstetrics and Gynecology, Touro College of Osteopathic Medicine, New York, USA

**Keywords:** fibroepithelial stromal polyp, giant acrochordon, labial mass, skin tag, vulvar mass

## Abstract

Acrochordons, also known as fibroepithelial stromal polyps or skin tags, are benign outgrowths of the skin that commonly occur in intertriginous regions and rarely grow larger than 1-5 mm in diameter. Benign acrochordons are common and can be difficult to distinguish from other benign stromal tumors and malignant growths. A 47-year-old woman with a body mass index of 26.6 kg/m² and no major predisposing factors presents with a giant acrochordon measuring 65 x 30 mm on the right labia majora. The lesion grew from 1 mm to 65 mm in approximately 18 months. The mass is associated with aesthetic dissatisfaction, itchiness, redness, and inner thigh friction that can lead to ulcerations. Histologic analysis is necessary for diagnostic confirmation. Aesthetic concerns and the importance of ruling out malignancy were factors that led to treatment with complete surgical excision.

## Introduction

An acrochordon is a small (1-5 mm), benign skin growth that is colloquially known as a skin tag or a fibroepithelial polyp. It is often found in skin fold areas, such as the neck or the axilla [1]. Although common on other body parts, large acrochordons in the genital area are extremely rare, with only a limited number of cases reported in the literature [2]. The pathogenesis of these large acrochordons may be associated with obesity, hormonal changes, or metabolic disorders [3]. They can occasionally reach sizes that cause discomfort or may cause aesthetic concerns for patients, prompting surgical intervention [4]. This report presents a case of a 47-year-old woman with an unusually large (65 mm) acrochordon located on the right labia majora, underscoring its unique presentation and history of growth. This case highlights the need for a diagnostic approach, immunopathological confirmation to exclude malignancies, and surgical management.

## Case presentation

**Figure 1:**
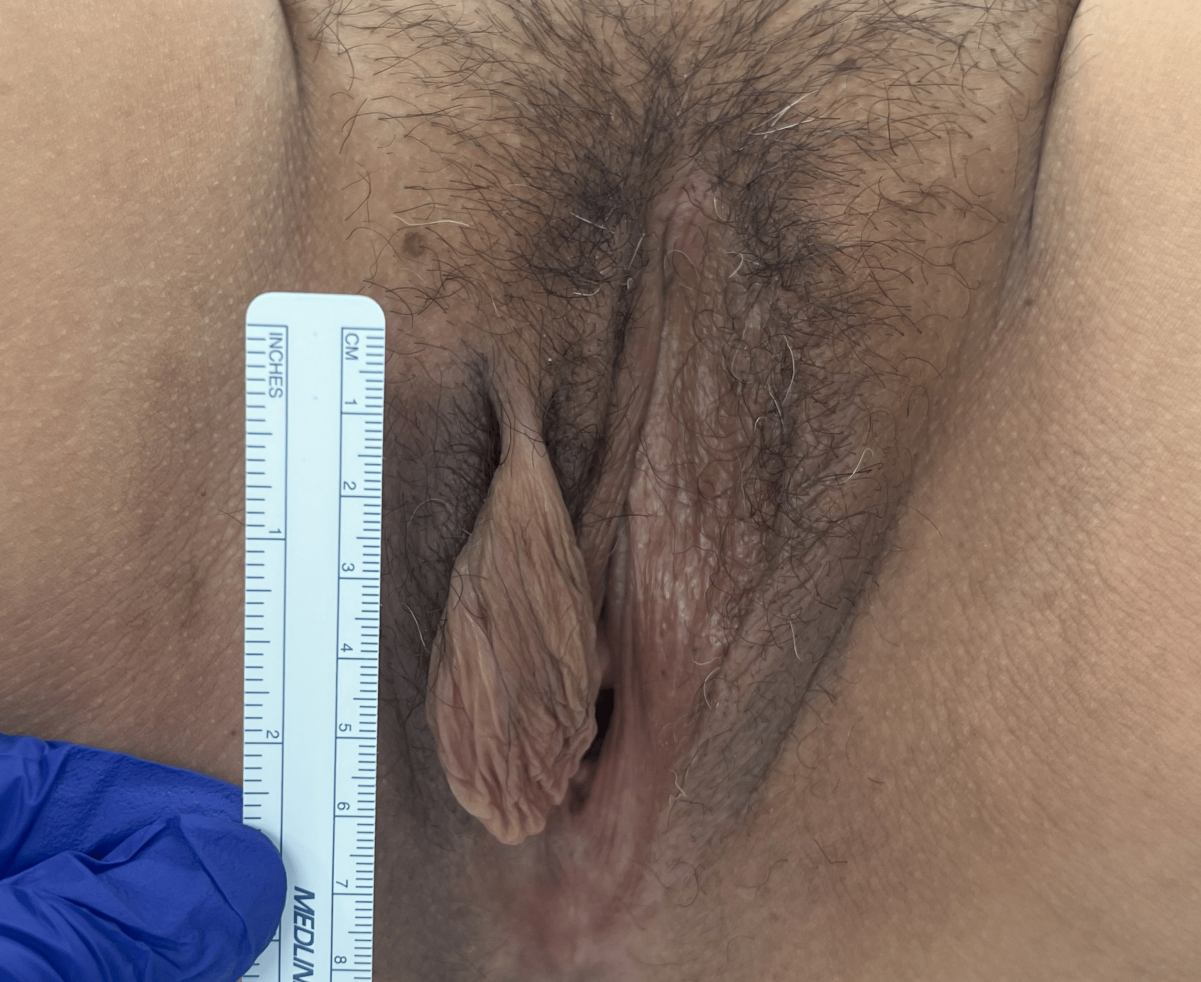
External view of the giant acrochordon on the right labia majora, measuring 65 x 30 mm The mass is flesh-colored, soft, and oblong in shape with a thin pedunculated attachment.

The patient, a 47-year-old woman, gravida 2 para 2, with a body mass index (BMI) of 26.6 kg/m², presented with a 65 x 30 cm acrochordon on her right labia majora. The mass appeared flesh-colored, soft, and oblong, with a thin, pedunculated attachment (Figure 1). The lesion had grown from 1 to 2 mm to 65 mm over 18 months. The patient delayed seeking medical attention due to embarrassment about the mass size. The mass was pruritic and red and caused friction with her inner thighs, leading to ulceration and a burning sensation. The patient denied vaginal pain or discharge.

Her past medical history is notable for endometrial polyps, endometriosis with uterine adhesions, ovarian cysts, and a left-sided breast mass. Her surgical history includes two cesarean sections, a dilation and curettage, a hysteroscopy, a cervical biopsy, a bilateral ovarian cystectomy, and a breast biopsy. The patient experienced menarche at 16 years old. The patient has regular menstrual cycles associated with heavy flows and dysmenorrhea. She denies a history of diabetes, hyperlipidemia, and sexually transmitted infections. She does not use hormonal contraceptives or other medications. Socially, the patient consumes alcohol but does not smoke and denies any illicit drug use. 

Operation report

The patient was taken to the operating room for excision of the right labial mass. She was positioned in lithotomy, and the empty sac was visualized hanging with a low tone and showing some degree of laxity. The mass was grasped and excised with a scalpel at its attachment to the labia. The incision extended into the greater labia majora base. There was no significant bleeding or discharge from the acrochordon or labial base.

**Figure 2:**
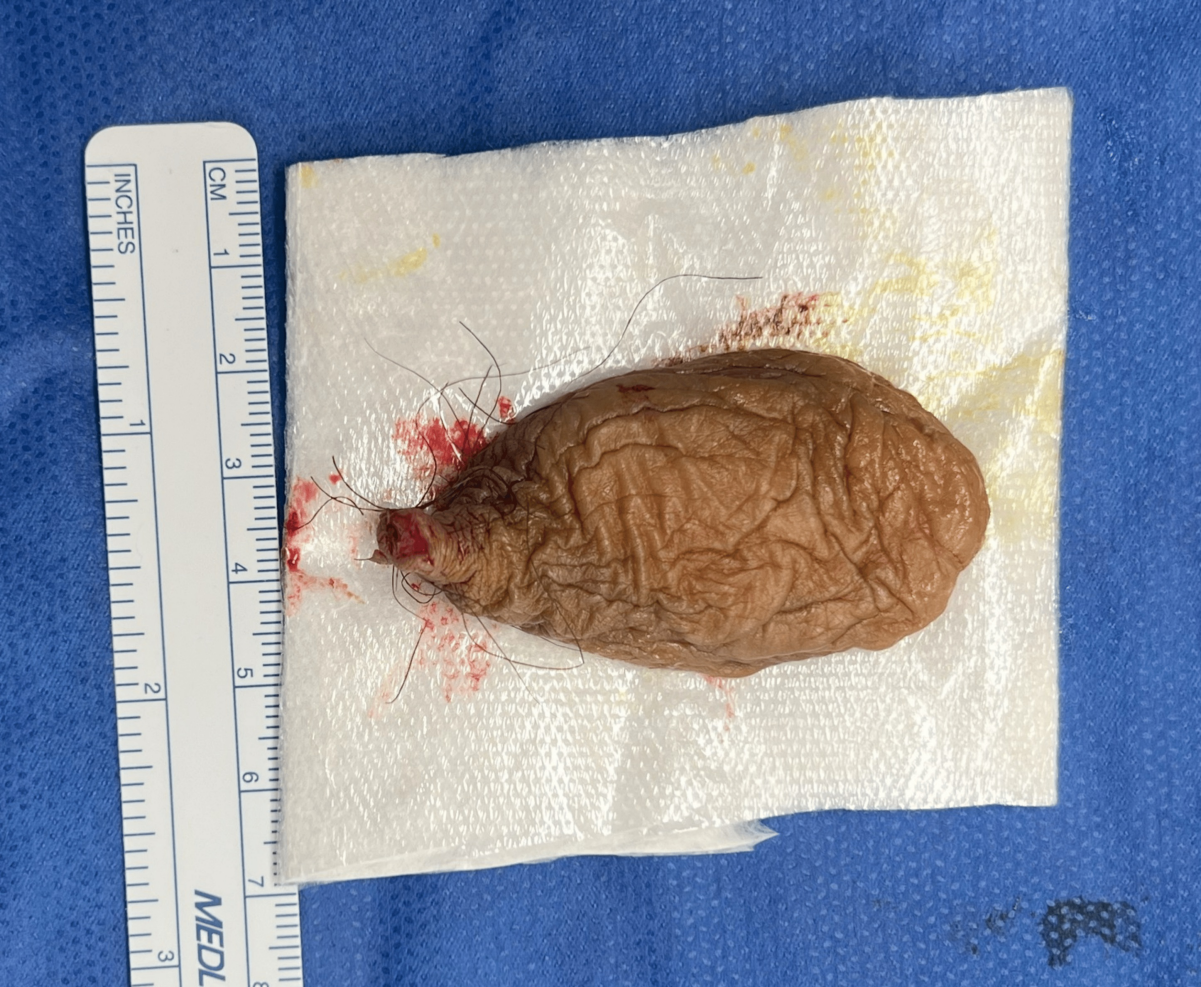
Measurement of the excised giant acrochordon after surgical removal, showing its size of approximately 65 x 30 mm The mass is characterized by thin, loose, redundant skin with minimal internal content. Measurement was made post-operatively with a surgical ruler (in mm).

After the excision, the mass was measured at approximately 65 x 30 mm (Figure 2). It was characterized as a flaccid, cutaneous sac composed of thin, loose, redundant skin with minimal internal content. The skin appeared wrinkled with a soft, collapsed appearance. The surface exhibited folds and creases due to reduced skin tension without significant underlying tissue. The incision site was closed with a single subcutaneous suture, and the remainder of the operative course was unremarkable.

**Figure 3:**
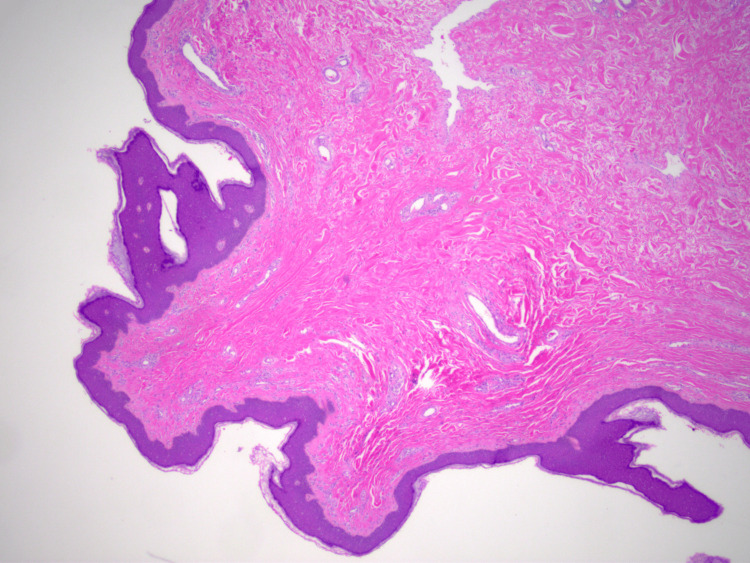
Hematoxylin and eosin (H&E) stain of the sectioned acrochordon The histological features include a well-defined, stratified epithelium-lined fibroconnective stroma with a core of fibroconnective tissue, confirming the benign nature of the lesion.

The excised mass was promptly sent to pathology, which showed a soft skin-covered polyploid mass. The specimen was serially sectioned to reveal a homogenous, soft, gray-white, and glistening tissue. A hematoxylin and eosin (H&E) stain revealed a well-defined, stratified epithelium-lined fibroconnective stroma with a core of fibroconnective tissue (Figure 3).

## Discussion

Acrochordons are also known as fibroepithelial polyps, soft fibromas, or skin tags. They are one of the most common benign dermatological neoplasms and are comprised of fibrous tissues from mesenchymal and ectodermal origins [5]. They are highly prevalent in the general population, with approximately 25-46% of people having one [1,2]. For female patients, genital acrochordons are most commonly found in the vagina [4]. The peak incidence of genital acrochordons is seen in women of reproductive age between 20 and 40 years of age [4]. They are rarely found in post-menopausal women [5].

Large acrochordons, particularly those in the genital region, are exceedingly rare. Giant acrochordons are at least 50 mm or more than 10x the size of an average acrochordon [5]. In the literature, a limited number of cases describe giant acrochordons of the labia majora, with their sizes ranging from 50 mm to 300 mm [2-10]. Giant acrochordons may cause patient discomfort, aesthetic concerns, or secondary complications such as friction-induced ulcerations or inflammation. They have also been associated with bleeding, dyspareunia, or vaginal discharge [10].

The pathogenesis of acrochordons remains poorly understood, although several factors contribute to their development. Associations have been established with obesity, endocrine disorders (such as diabetes mellitus and insulin resistance), genetics/family disposition, lipid and carbohydrate metabolism, and hormonal changes [3]. It has been hypothesized that acrochordons occur in areas with less elastic tissue, which results in them manifesting as atrophic or sessile masses [1]. One paper found that higher numbers of mast cells were associated with developing skin tags because mast cells attracted eosinophils, which in turn promote and induce excessive fibrosis [11]. Hormonal changes, such as high levels of progesterone and estrogen during pregnancy, have been linked to the development of acrochordons due to the proliferation of mesenchymal cells within the hormonally sensitive subepithelial stromal layer of the lower genital tract [4]. Lastly, an analysis by Dianzni, et al. found that 71-88% of skin tags had human papillomavirus infections [12].

Histologically, giant pedunculated acrochordons often show a flat squamous epidermis layer on top, an intermediate layer with loose collagen fibers, and a core with mature fat cells [5,13]. Acrochordons also display a stromal component with prominent cellularity and a central vascular core. Hypocellular regions appear myxoid and edematous, containing scattered spindle cells, while hypercellular areas feature multinucleate or stellate cells. These stromal cells demonstrate immunoreactivity to estrogen and progesterone receptors, desmin, and smooth muscle actin [5,14].

The differential diagnosis of large vulvar acrochordons includes a range of benign and malignant masses. Among the benign entities, a few differential diagnoses include Bartholin cysts, hernias, lipomas, and hemangiomas. Another benign tumor that can present similarly is a canal of Nuck tumor, a soft tissue tumor that forms from inadequate closure of the processus vaginalis [6]. Malignant lesions are more critical to exclude due to their propensity for local invasion and recurrence. A few particular malignancies to consider are aggressive angiomyxoma, angiomyofibroblastoma, botryoid embryonal rhabdomyosarcoma, and neurofibromas [4]. In addition, there have been instances of squamous cell carcinoma and basal cell carcinoma associated with cutaneous skin tags [7,8]. Imaging, such as an MRI, may help distinguish these lesions by assessing vascular patterns and the extent of growth. A biopsy and histopathologic examination after a mass’s surgical excision are essential to rule out differential diagnoses [3,4,5,7]. 

The definitive treatment for a giant acrochordon is complete surgical excision or laser ablation, which ensures both diagnosis of the mass and resolution of symptoms, particularly those that cause discomfort or have ulceration. Incomplete removal may leave residual tissue that can regrow; therefore, long-term follow-up should be conducted to observe for recurrences as soon as possible [2]. 

Acrochordons are most commonly observed in obese women of reproductive age, particularly during pregnancy, suggesting a possible hormonal influence. They are often associated with symptoms such as bleeding, dyspareunia, and vaginal discharge. This patient’s case is unique in both history and presentation. Unlike other reported cases, this patient was overweight (BMI 26.6 kg/m²) but not obese. Moreover, she had no chronic medical conditions, such as insulin-resistant diabetes or hyperlipidemia. She was not using hormonal therapy or contraceptives, and there were no recent pregnancies that may have resulted in any hormonal changes. Additionally, she denied experiencing bleeding, dyspareunia, or discharge; her primary concern was occasional discomfort. Notably, her vulvar acrochordon grew rapidly, increasing from 1 mm to 65 mm over 18 months.

## Conclusions

A giant acrochordon of the labia majora is a rare presentation of vulvar skin tags that exceed 50 mm in size. Oftentimes, they start as 1-5 mm but may expand. Physicians should monitor skin tags on the labia majora in all female patients because they can enlarge, on average, over two years. Ulceration, discomfort, and aesthetic concerns may complicate the presentation of giant acrochordons. For these psychosocial reasons, patients may be reluctant to visit a gynecologist to have an acrochordon evaluated. The standard of care for large acrochordons is surgical excision or laser ablation with vulvar reconstruction. This report details a rare case of a 47-year-old woman with a large 65 x 30 mm acrochordon located on her right labia majora. Treatment with surgical excision and vulvar reconstruction was successfully conducted under general anesthesia. During her two-week follow-up appointment, the patient was recovering without complications.
